# Is radical local therapy effective in postoperative recurrent *EGFR* ‐mutated non‐small cell lung cancer?

**DOI:** 10.1111/1759-7714.14911

**Published:** 2023-05-04

**Authors:** Tomoyoshi Takenaka, Tokujiro Yano, Koji Yamazaki, Tatsuro Okamoto, Motoharu Hamatake, Shinkichi Takamori, Mikihiro Kohno, Naoko Miura, Mototsugu Shimokawa, Tomoharu Yoshizumi, Kyushu University Lung Surgery Study Group

**Affiliations:** ^1^ Department of Surgery and Science, Graduate School of Medical Sciences Kyushu University Fukuoka Japan; ^2^ Department of General Thoracic Surgery National Hospital Organization, Beppu Medical Center Beppu Japan; ^3^ Department of Thoracic Surgery and Clinical Research Institute National Hospital Organization, Kyushu Medical Center Fukuoka Japan; ^4^ Department of Thoracic Oncology National Kyushu Cancer Center Fukuoka Japan; ^5^ Department of Thoracic Surgery Kitakyushu Municipal Medical Center Kitakyushu Japan; ^6^ Department of Biostatistics Yamaguchi University Graduate School of Medicine Ube Japan

**Keywords:** epidermal growth factor receptor mutation, non‐small cell lung cancer, post‐recurrence survival, recurrence, surgical resection

## Abstract

**Background:**

Long‐term survival can be achieved with radical local therapy in some cases of postoperative recurrence of non‐small cell lung cancer (NSCLC). Here, we evaluated post‐recurrence survival (PRS) after treatment of postoperative recurrent epidermal growth factor receptor (*EGFR*) mutated NSCLC and examined the effectiveness of radical local therapy.

**Methods:**

This multicenter prospective cohort study was conducted in 14 hospitals. The inclusion criteria for this study were patients with recurrence after radical resection for NSCLC. Information about the patient characteristics at recurrence, tumor‐related variables, primary surgery, and treatment for recurrence was collected. After registration, follow‐up data (e.g., treatment and survival outcomes) were obtained and analyzed.

**Results:**

From 2010 to 2015, 505 patients with recurrent NSCLC were enrolled into the study, and 154 *EGFR* mutation‐positive cases were included. As the initial treatment for recurrence, 111 patients (72%) received chemotherapy, 14 (9%) received chemoradiotherapy, 14 (9%) received definitive radiotherapy, and seven (5%) received surgical resection. The remaining eight patients (5%) received supportive care. The median PRS and 5‐year survival rates for all cases were 64 months and 53.2%, respectively. The 5‐year survival rate according to the initial treatment was as follows: supportive care, 0%; chemotherapy, 53.3% and radical local therapy, 60.1%. The six patients who received radical local treatment remained recurrence‐free for more than 3 years after recurrence with only initial treatment.

**Conclusions:**

Although radical local therapy may be curative in some patients, chemotherapy including EGFR‐TKI treatment is expected to provide long‐term survival comparable to that of radical local therapy.

## Graphical abstract

This study evaluated 154 patients with recurrent non‐small cell lung cancer (NSCLC) with epidermal growth factor receptor (*EGFR*) mutation who were prospectively registered at multiple institutions. In particular, we examined the efficacy of radical local therapy in patients with postoperative recurrent *EGFR* mutated NSCLC.

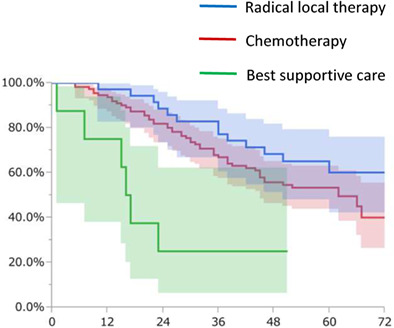

## INTRODUCTION

Approximately 2.0 million incident cases of lung cancer and 1.7 million deaths worldwide have been reported, and lung cancer continues to be the most common type of cancer.^1^ Surgery is the best therapeutic modality for patients with early‐stage non‐small cell lung cancer (NSCLC). Although the recurrence rate varies depending on the stage, it is reported to be 20%–50%, even with recent advances in postoperative adjuvant chemotherapy.^2^, ^3^, ^4^, ^5^, ^6^, ^7^, ^8^

Treatment for recurrent disease is usually similar to that used for advanced diseases. According to the National Comprehensive Cancer Network guidelines for NSCLC, surgery and radiation therapy are recommended for resectable local recurrence, and concurrent chemoradiotherapy is recommended for mediastinal lymph node metastasis.^9^ In contrast, systemic therapy is recommended for distant metastasis, regardless of the involved organ (excluding the brain and bone) and the number of recurrent foci.^9^

Thoracic oligo‐recurrence of NSCLC has been reported to show a favorable outcome in a select population.^10^, ^11^, ^12^, ^13^, ^14^, ^15^, ^16^ While there is currently no clear consensus concerning the most appropriate treatment, local therapy, such as surgery, radiotherapy, or chemoradiotherapy, may be a common treatment option.^10^, ^11^, ^12^, ^13^, ^14^, ^15^, ^16^ In addition, some patients who receive appropriate local therapy—even those with recurrent disease—may obtain a cure.

Epidermal growth factor receptor (*EGFR*) is a receptor kinase that is highly expressed in cancer cells. *EGFR* mutations such as exon 19 deletions and exon 21 L858R point mutations, are common oncogenic driver mutations in NSCLC.^17^, ^18^ EGFR‐tyrosine kinase inhibitors (EGFR‐TKIs), such as gefitinib, erlotinib, afatinib, dacomitinib and osimertinib have been used to treat NSCLC, and extremely good responses have been achieved in *EGFR* mutated NSCLC patients.^19^ While the efficacy of EGFR‐TKI has been reported for cases of postoperative recurrent cases of *EGFR*‐positive NSCLC, there are few reports on the outcomes of radical local therapy.

In this study, a prospective multicenter cohort study was designed to evaluate the post‐recurrence survival (PRS) of patients with recurrent NSCLC. In particular, we examined the efficacy of radical local therapy in patients with postoperative recurrent *EGFR* mutated NSCLC.

## METHODS

### Patients

The present trial was planned by the Kyushu University Lung Surgery Study Group, started in July 2010, and was conducted in 14 hospitals in Japan. This study was a prospective observational study to evaluate the PRS after treatment of recurrent NSCLC, to evaluate the PRS according to the initial treatment for recurrent disease, and to identify the prognostic factors associated with PRS. Patients with recurrence after radical resection for NSCLC were enrolled in this study. A total of 505 patients with recurrent NSCLC were enrolled in this study, which was completed on March 31, 2021. Written informed consent was obtained from each patient according to the regulations of each participating institution. This study was approved by the institutional review board of Kyushu University Hospital (IRB no.: 21–152; date approved: March 31, 2010).

### Perioperative examination

The preoperative diagnosis was determined based on the findings of chest and upper abdomen computed tomography (CT), brain CT or magnetic resonance imaging (MRI), radionuclide bone scans, and/or fluorodeoxyglucose‐positron emission tomography (FDG‐PET). The histological diagnosis of the tumors was classified according to the World Health Organization (WHO) classification of lung and pleural tumors in 1999 or the 2015 WHO Classification of Tumors of the Lung, Pleura, Thymus, and Heart.^20^, ^21^

Postoperative follow‐up examinations were performed according to the policy of each hospital. Generally, follow‐up examinations are usually conducted every 3–4 months for the first 2 years and every 3–6 months thereafter. Routine follow‐up procedures included physical examinations, hematological examination, and chest radiography. In addition, chest and abdominal CT were performed at least once per year. If recurrent disease was suspected, further evaluations, such as MRI and FDG‐PET, were added. Recurrent NSCLC was diagnosed based on the findings consistent with recurrent disease on a physical examination and diagnostic imaging. When clinically feasible, the diagnosis was histologically confirmed. The date of recurrence was defined as recurrence that was histologically proven or—in cases diagnosed based on clinical evidence—when recurrent disease was recognized by the attending physician. The disease‐free interval was defined as the period from the date of surgery to the date on which recurrence was detected. Local recurrence was defined as disease recurrence at the surgical margin, ipsilateral hemithorax, or mediastinum. Ipsilateral hilar and mediastinal lymph node recurrence were defined as local recurrence. The recurrence in a separate lobe in the ipsilateral hemithorax was also defined as local recurrence. Distant metastasis was defined as disease recurrence in the contralateral lung or outside the hemithorax and mediastinum. A second lung tumor that was diagnosed as a different cell type from the first tumor before treatment was excluded. In cases where there was no histological diagnosis, although solitary pulmonary nodules that was met the Martini and Melamed criteria for second primary lung cancer (e.g., tumor without regional lymph node metastasis or distant metastasis that appeared more than 2 years after the first surgery),^22^ nodules that the attending physician judged to be postoperative recurrence were included in this study. The definition of oligo‐recurrence in this study was a case with no more than three recurrent foci regardless of the number of involved organs according to diagnostic imaging. The treatment policy for recurrence was left to each hospital. The patient selection flow chart is shown in Figure SS1. In this study, surgery, chemoradiotherapy, and radiation therapy were defined as radical local therapy.

### Data collection and extraction

For each patient, we collected the following information at the time of registration: (i) general characteristics at recurrence, (ii) tumor‐related variables, (iii) information on surgery, and (iv) treatment for recurrence. The general characteristics at recurrence included age, sex, Eastern Cooperative Oncology Group (ECOG) performance status (PS), symptoms of recurrence, physical findings, and the data on which recurrence was confirmed. Tumor‐related variables included histological type, recurrent organs, site of recurrence, number of recurrent foci, and *EGFR* mutation status. Information on surgery included the date of surgery, type of resection, pathological stage, and presence of postoperative adjuvant chemotherapy. Treatment for recurrence included the date on which treatment for recurrence was initiated, the initial treatment for recurrence, and the chemotherapy regimen. After registration, follow‐up data, such as the outcome of treatment, change of treatment, and survival outcome, were obtained every 3 months.

### Statistical analysis

Comparisons of dichotomous variables between groups were performed using the *χ* ^2^‐test. Post‐recurrence survival was measured from the date of initial recurrence to the date of death from any cause or the date on which the patient was last known to be alive. Survival probability was estimated using the Kaplan–Meier method. Differences in survival were evaluated using log‐rank tests. Univariate and multivariate analyses associated with PRS were tested using a Cox proportional hazards regression model. Analyses were performed using the JMP software package (version 11; SAS Institute Inc., USA). *p*‐values of <0.05 were considered to indicate statistical significance.

## RESULTS

The characteristics of the patients at the time of recurrence are summarized in Table 1 and a summary of this study is shown in Figure SS1. A total of 154 patients were analyzed in this study, including 57 men (37%) and 97 women (63%). The median age of the patients was 71 years (range, 32–98 years), and 144 (90%) had an ECOG PS of 0 or 1 at the time of initial recurrence. Adenocarcinoma was the most common type of cancer in (*n* = 152); the other two cases were other histological types. A total of 132 patients underwent lobectomy, six underwent pneumonectomy, and 16 underwent sublobar resection as primary surgery. The median disease‐free interval from surgery to the detection of the first recurrence was 21 months (range, 2–158 months). Among the 154 patients, 51 (33%) had local recurrence and 104 (67%) had distant recurrence. A total of 69 cases had *EGFR* exon 19 deletions, 71 had exon21 L858R mutations, and 14 had other mutations. The most commonly involved organs were the lung (*n* = 70), followed by the hilar and mediastinal lymph nodes (*n* = 54), bone (*n* = 29), brain (*n* = 26), pleura (*n* = 23), liver (*n* = 2), adrenal grand (*n* = 2) and other organs (*n* = 14) (Table 1).

**TABLE 1:** Patient characteristics.

Variable	*n* = 154
Age (range) (years)	71 (32–98)
Sex	
Male	57 (37%)
Female	97 (63%)
Performance status	
0	102 (66%)
1	42 (27%)
2, 3	10 (7%)
Histological type	
Adenocarcinoma	152 (99%)
Others	2 (1%)
Primary operation	
Lobectomy	132 (86%)
Pneumonectomy	6 (4%)
Sublobar resection	16 (10%)
Pathological stage at initial surgery	
Stage I	71 (46%)
Stage II	35 (23%)
Stage III	48 (31%)
Symptoms at recurrence	
With symptoms	34 (22%)
Without symptoms	120 (78%)
Site of recurrence	
Distant recurrence	103 (67%)
Local recurrence	51 (33%)
Time to recurrence	
<1 year	56 (36%)
≥1 year	98 (54%)
Number of recurrent foci	
1	46 (30%)
2	22 (14)
3	11 (7%)
4 or more	75 (49%)
*EGFR* mutation status	
Exon 19	69 (45%)
Exon 21	71 (46%)
Others	14 (9%)
Recurrent organs	
Lymph node	54 (35%)
Lung	70 (45%)
Bone	29 (19%)
Pleura	23 (15%)
Brain	26 (17%)
Liver	2 (1%)
Adrenal grand	2 (1%)
Other	14 (9%)

Abbreviation: *EGFR*, epidermal growth factor receptor.

As initial treatment for recurrence, 111 patients (72%) received chemotherapy, 14 (9%) received chemoradiotherapy, 14 (9%) received definitive radiotherapy, 14 (9%) and seven (5%) received surgical resection. EGFR‐TKI was administered to 104 patients. All 104 patients received first‐ or second‐generation EGFR‐TKIs such as gefitinib, erlotinib or afatinib. The remaining eight patients (5%) received supportive care only (Table 2). The relationship between the recurrence patter and initial treatment are shown in Table 2. Patients with local recurrence more often received local treatment (e.g., surgery, chemoradiotherapy, or definitive radiotherapy), whereas patients with distant recurrence were more often treated with chemotherapy (Table 2). Patients with oligo‐recurrence were more often treated with radical local therapy (e.g., surgery, chemoradiotherapy, or definitive radiotherapy), whereas most cases of multiple recurrence were treated with chemotherapy (Table 3). The chemotherapy regimens of the chemotherapy group included cytotoxic drugs (*n* = 7) and EGFR‐TKIs (*n* = 104). No patient received immune checkpoint inhibitors as an initial treatment. Chemotherapy regimens in the chemoradiotherapy group included cytotoxic drugs (*n* = 10) and EGFR‐TKIs (*n* = 4).

**TABLE 2:** Initial treatment according to the pattern of recurrence.

Treatment		Pattern of recurrence		
	*N* (%)	Local recurrence	Distant recurrence	*p*‐value
Supportive care only	8 (5%)	3 (38%)	5 (62%)	<0.001
Chemotherapy	111 (72%)	32 (29%)	79 (71%)	
Radical local therapy	35 (23%)	19 (54%)	16 (46%)	
Surgery	7 (5%)	5 (71%)	2 (29%)	
Chemoradiotherapy	14 (9%)	9 (64%)	5 (36%)	
Radiotherapy	14 (9%)	5 (36%)	9 (64%)	

**TABLE 3:** Initial treatment according to the number of recurrent foci.

Treatment		Number of recurrent foci		
	*N* (%)	Oligo‐recurrence	Multiple recurrence	*p*‐value
Supportive care only	8 (5%)	3 (38%)	5 (62%)	<0.001
Chemotherapy	111 (72%)	44 (40%)	67 (60%)	
Radical local therapy	35 (23%)	32 (91%)	3 (9%)	
Surgery	7 (5%)	7 (100%)	0 (0%)	
Chemoradiotherapy	14 (9%)	11 (79%)	3 (21%)	
Radiotherapy	14 (9%)	14 (100%)	0 (0%)	

**FIGURE 1:**
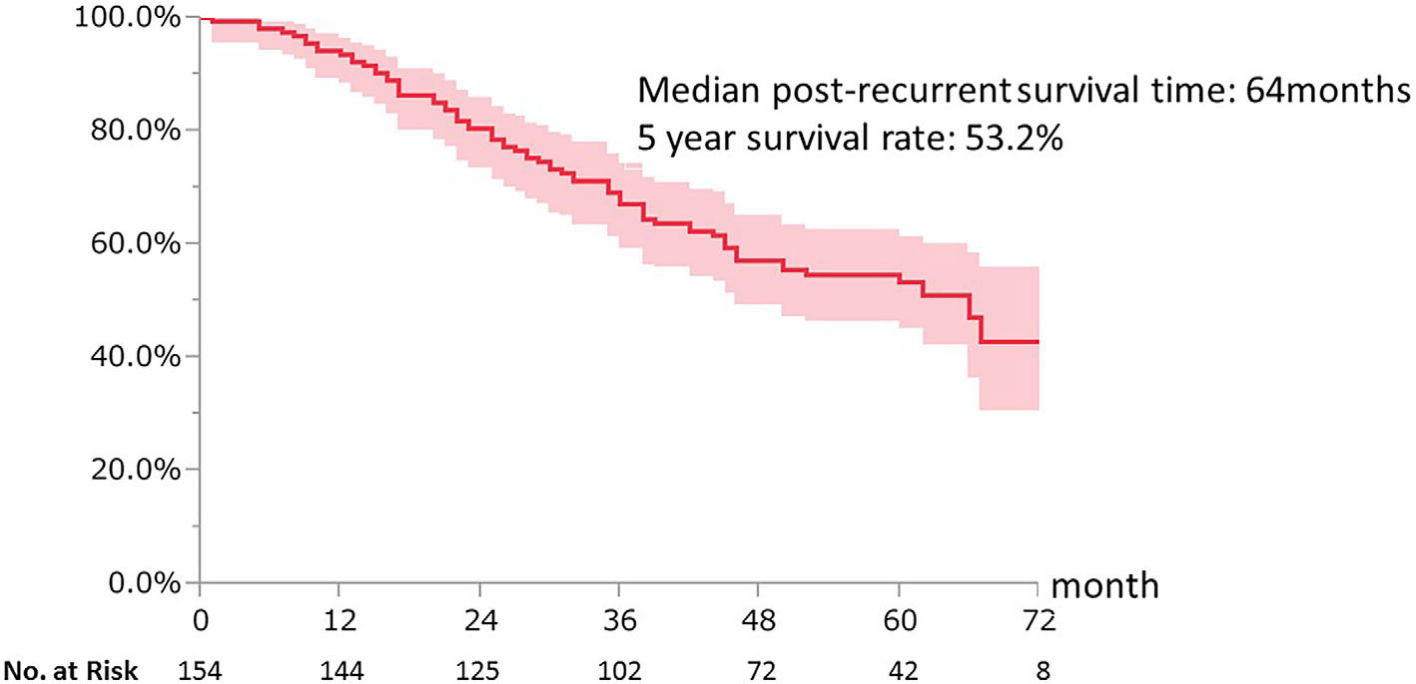
Kaplan–Meier curves of post‐recurrence survival of all cases. The filled area represents the 95% confidence interval (CI).

**FIGURE 2:**
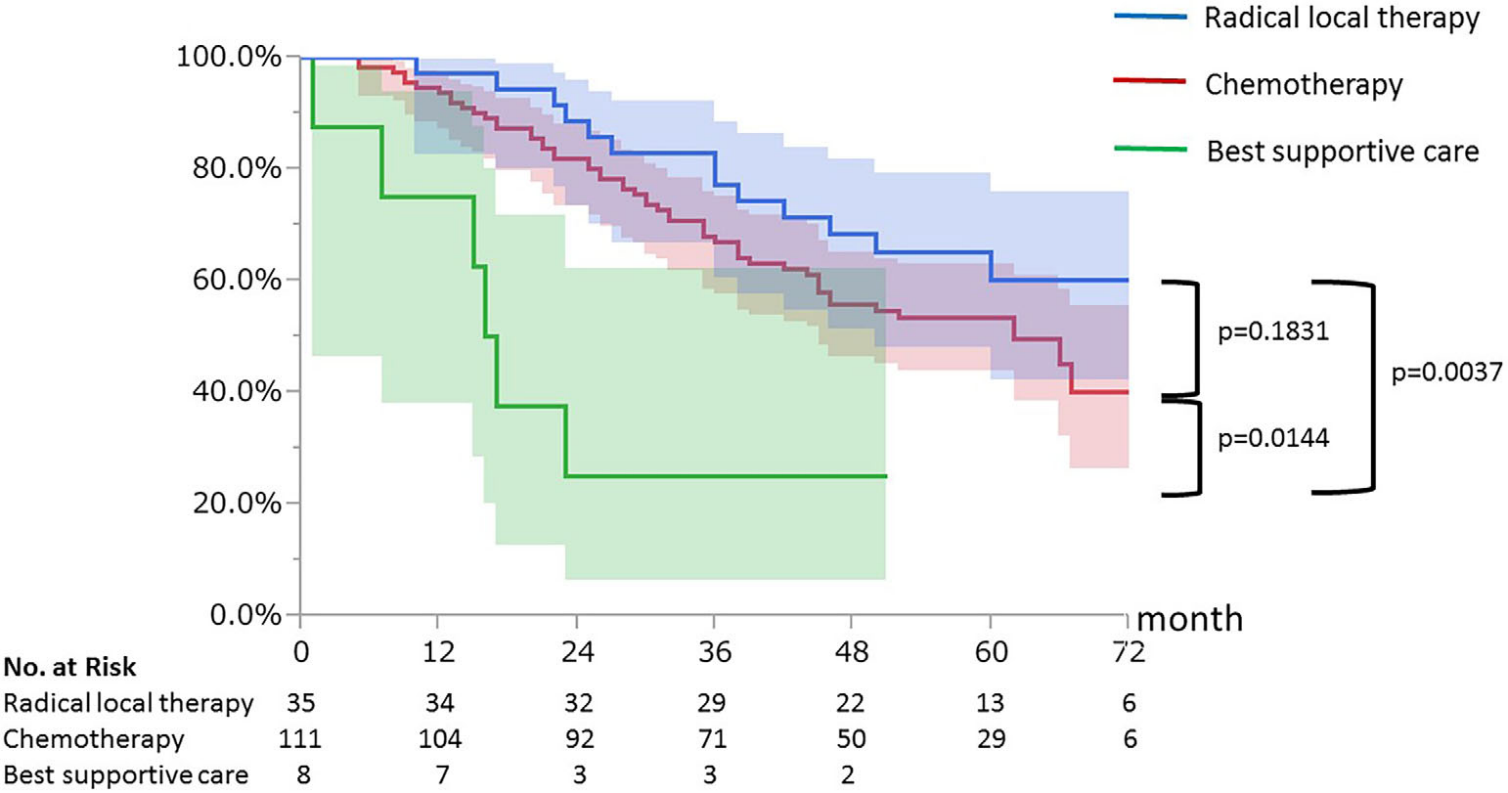
Kaplan–Meier curves of post‐recurrence survival according to the initial treatment. The filled area represents the 95% confidence interval (CI).

The median follow‐up period of survivors was 59 months (range 7–89 months). The median PRS and 5‐year survival rates of all patients were 64 months (range, 2–89 months) and 53.2%, respectively (Figure 1). The median PRS and 5‐year PRS rates according to the initial treatment were as follows: supportive care, 18 months and 0%; chemotherapy, 61 months and 53.3% and radical local therapy, not reached, and 60.1%, respectively (Figure 2). The six patients who received radical local therapy achieved recurrence‐free survival for more than 3 years after recurrence with only initial treatment. Radical local therapy tended to be better than chemotherapy for survival after recurrence, but the difference was not statistically significant (*p* = 0.1831). The median PRS according to the type of mutation was 62 months for *EGFR* exon 19 deletions, 66 months for exon21 L858R mutations, and 40 months for other mutations.

A univariate analysis showed that PS, presence of symptoms at recurrence, number of recurrent foci, and initial treatment for recurrence influenced the PRS (Table 4). A multivariate analysis showed that PS, presence of symptoms at recurrence, and number of recurrent foci independently influenced the PRS (Table 4).

**TABLE 4:** Univariate and multivariate analyses of factors predicting post‐recurrence survival.

	Univariate		Multivariate	
	HR (95% CI)	*p*‐value	HR (95% CI)	*p*‐value
Age				
≥75	1.408 (1.265–1.974)	*p* = 0.155		
75	1.0			
Sex				
Female	1.104 (0.687–1.814)	*p* = 0.688		
Male	1.0			
Performance status				
PS ≥2	4.643 (2.036–9.227)	*p* < 0.001	5.381 (2.142–12.276)	*p* < 0.001
PS 0 or 1	1.0		1.0	
Histology				
Nonadenocartinoma	2.153 (0.121–9.916)	*p* = 0.500		
Adenocarcinoma	1.0			
Primary operation				
Standard resection	1.151 (0.566–2.762)	*p* = 0.719		
Sublobar resection	1.0			
Adjuvant chemotherapy				
Yes	1.097 (0.690–1.747)	*p* = 0.695		
No	1.0			
Symptoms at recurrence				
With symptoms	2.436 (1.451–3.975)	*p* = 0.001	1.748 (1.020–2.919)	*p* = 0.043
Without symptoms	1.0		1.0	
Recurrent site				
Distant	1.527 (0.920–2.645)	*p* = 0.103		
Local	1.0			
Time to recurrence				
<1 year	1.583 (0.987–2.520)	*p* = 0.057		
≥1 year	1.0			
Number of recurrent foci				
≥4	2.153 (1.344–3.516)	*p* = 0.001	2.599 (1.446–4.849)	*p* = 0.001
1–3	1.0		1.0	
Pathological stage				
≥Stage II	1.482 (0.929–2.399)	*p* = 0.100		
Stage I	1.0			
Initial treatment for recurrence				
Supportive care only	5.156 (1.798–13.181)	*p* = 0.014	1.870 (0.581–5.419)	*p* = 0.240
Chemotherapy	1.488 (0.837–2.851)		0.829 (0.411–1.745)	
Radical local therapy	1.0		1.0	

Abbreviations: CI, confidence interval; HR, hazard ratio; PS, performance status.

## DISCUSSION

Thoracic oligo‐recurrence of NSCLC has been reported to show a favorable outcome in a select population.^10^, ^11^, ^12^, ^13^, ^14^, ^15^, ^16^ While there is currently no clear consensus concerning the most appropriate treatment, local therapy (e.g., surgery, radiotherapy, or chemoradiotherapy) may be a promising treatment option.^10^, ^11^, ^12^, ^13^, ^14^, ^15^, ^16^ In addition, some cases of recurrent disease are curatively treated by local therapy. On the other hand, there are few reports on the efficacy of radical local therapy in patients with recurrent NSCLC who are positive for *EGFR* mutations, as EGFR‐TKIs enable long‐term survival in this patient group.

In this study, we prospectively evaluated the post‐recurrence outcomes of patients with recurrence after curative resection for NSCLC. The median PRS and 5‐year survival rates of 154 *EGFR* mutation‐positive patients were 64 months (range, 2–89 months) and 53.2%, respectively. In Japan, the median PRS of recurrent NSCLC was reported to be 17.7–25 months (2–4); thus, it was shown that *EGFR* mutation‐positive patients were expected to achieve very long survival, even if they developed postoperative recurrence.

In this study, 104 patients received EGFR‐TKIs as first‐line chemotherapy. All patients received first‐ or second‐generation EGFR‐TKIs. Takenaka et al. reported that the median post‐recurrence survival time according to the use of EGFR‐TKI therapy was 49 months in *EGFR* mutation‐positive patients in a study performed in 2000 to 2001.^4^ In our study, which accumulated cases from the 2010s, treatment outcomes have improved. One reason for the improved outcomes is the subsequent development of new drugs.

Osimertinib is a third‐generation, irreversible EGFR‐TKI that selectively inhibits both EGFR‐TKI‐sensitizing and *EGFR* T790M resistance mutations. Ramlingam et al. reported the efficacy of osimertinib in comparison to first‐generation EGFR‐TKIs in 2020.^23^ According to the report, the median overall survival was 38.6 months in the osimertinib group and 31.8 months in the comparator group in which patients received first generation EGFR‐TKIs such as gefitinib or erlotinib. Based on these results, osimertinib is now the first‐line drug for patients with previously untreated advanced NSCLC with an *EGFR* mutation.^23^ In this study, no patient received osimertinib as first line therapy after recurrence, but there were patients who received it in second‐line or subsequent therapy.

In this study, we also examined the efficacy of radical local therapy (e.g., surgery, definitive radiotherapy, and chemoradiotherapy) for *EGFR* mutated recurrent NSCLC. Although the treatment method was not clearly defined for each pattern of recurrence, radical local therapy was often chosen for local recurrence. In addition, in most cases, radical local therapy was administered for patients with oligo‐recurrence. The median PRS and 5‐year PRS rates according to the initial treatment were as follows: supportive care, 18 months and 0%; chemotherapy, 61 months and 53.3% and radical local therapy, not reached, and 60.1%, respectively. Radical local therapy tended to be better than chemotherapy for survival after recurrence, but the difference was not statistically significant. On the other hand, the six patients who received radical local therapy remained recurrence‐free survivors for more than 3 years after recurrence with only initial treatment. These patients achieved long treatment‐free periods after radical local therapy. In some cases, recurrent NSCLC may have been curatively treated. In fact, the efficacy of radical local therapy for recurrent NSCLC has long been reported.^10^, ^11^, ^12^, ^13^, ^14^, ^15^, ^16^, ^24^ Matsuguma et al. reported that that recent recurrence, oligo‐recurrence, and radical local therapy were associated with an improved median PRS time and long‐term PRS rate in patients with postoperative recurrence after complete resection of NSCLC.^12^ Takenaka et al. reported the usefulness of concurrent chemoradiotherapy for recurrent NSCLC in select patients.^24^ They found that 20% of patients who received concurrent chemoradiotherapy for local recurrence remained disease‐free for more than 3 years after chemoradiotherapy.^24^

Oligo‐recurrence is usually defined as ≤3–5 or less distant metachronous metastases that can be treated with local therapy with controlled primary lesions.^10^, ^11^, ^12^, ^13^, ^14^, ^15^, ^16^ Although the role of radical treatment for oligo‐recurrence is not well established, good PRS was reported in the subgroup of patients who received radical therapy (e.g., surgery or definitive radiotherapy).^10^, ^11^, ^12^, ^13^, ^14^, ^15^, ^16^ Indeed, the multivariate analysis of this study showed that patients with oligo‐recurrence had a significantly better PRS than those with multiple recurrence. Seol et al. evaluated the clinical outcomes of salvage radiotherapy for patients with lymph node oligo‐recurrence that developed after radical surgery for NSCLC. They reported that the 1‐ and 2‐year recurrence‐free survival rates were 73.1% and 50.9%, respectively.^14^ Han et al. reported the outcome of pulmonary oligo‐recurrence (≤5 metastatic lesions). They compared the PRS of patients who received operative or nonoperative treatment, including chemotherapy, radiotherapy, chemoradiotherapy, and best supportive care.^15^ The 5‐year PRS rates in the operative and nonoperative groups were 67% and 26%, respectively. They concluded that operative treatment of pulmonary oligo‐recurrence significantly prolonged PRS in patients who underwent curative resection for NSCLC.^15^ Aoki et al. reported the outcome of salvage stereotactic body radiotherapy (SBRT) for oligo‐recurrence of NSCLC. The median OS following salvage SBRT was 32 months, and the 1‐ and 3‐year OS rates were 84.4% and 67.8%, respectively.^16^ In the present study, although 79 patients (51%) of the patients had oligo‐recurrence, only 32 patients (20%) received radical local therapy. The results suggest that EGFR‐TKIs may be administered in many cases, even for oligo‐recurrence, due to the high therapeutic efficacy.

The present study was associated with several limitations. First, since this study was observational rather than interventional, registration was not based on strict definitions (e.g., histological or genetic diagnosis of recurrence). The fact that the diagnosis of recurrence was made according to the criteria of each institution cannot be denied. The treatment policy was also not shared among institutions. As the diagnosis of recurrence, indications, and therapeutic strategies for recurrent disease were generally examined according to the standard of each institution, not all patients received treatment according to the same standard. Second, the PRS may be regulated not only by the initial therapy but also by the second‐ or third‐line therapy. Indeed, some patients received new anticancer drugs (e.g., third generation EGFR‐TKI and immune checkpoint inhibitors), while others did not. Furthermore, since this was an observational study, the timing of treatment unavoidably had an effect. Despite these limitations, this study evaluated 154 patients with recurrent NSCLC with *EGFR* mutation who were prospectively registered at multiple institutions.

In conclusion, the prognosis of postoperative recurrence of *EGFR*‐mutant NSCLC was improved by chemotherapy and radical local therapy. Whenever possible, aggressive anticancer therapy should be used instead of best supportive care alone. Although radical local therapy may be curative in some patients, chemotherapy including EGFR‐TKIs is expected to provide long‐term survival comparable to that of radical local therapy. More clinical studies are needed to establish a treatment method based on the condition of recurrence.

## AUTHOR CONTRIBUTIONS

Tomoyoshi Takenaka: Validation; formal analysis; writing–original draft preparation. Tokujiro Yano: Conceptualization; methodology; writing–review and editing preparation. Koji Yamazaki: Data curation; investigation. Tatsuro Okamoto: Data curation; investigation. Motoharu Hamatake: Data curation; investigation. Shinkichi Takamori: Data curation; investigation. Mikihiro Kohno: Data curation; investigation. Naoko Miura: Data curation; investigation. Mototsugu Shimokawa: Software; formal analysis; project administration. Tomoharu Yoshizumi: Supervision.

## CONFLICT OF INTEREST STATEMENT

The authors declare no conflicts of interest.

## INFORMED CONSENT STATEMENT

Written informed consent was obtained from each patient according to the regulations of each participating institution.

## Supporting information


**Figure S1.** Consort diagram of the study.

## Data Availability

The data underlying this article will be shared on reasonable request to the corresponding author.

## References

[1] Fitzmaurice C , Abate D , Abbasi N , Abbastabar H , Abd‐Allah F , Abdel‐Rahman O , et al. Global, regional, and national cancer incidence, mortality, years of life lost, years lived with disability, and disability‐adjusted life‐years for 29 cancer groups, 1990 to 2016: a systematic analysis for the global burden of disease study. JAMA Oncol. 2018;4:1553–68. 29860482 10.1001/jamaoncol.2018.2706 PMC6248091

[2] Saisho S , Yasuda K , Maeda A , Yukawa T , Okita R , Hirami Y , et al. Post‐recurrence survival of patients with non‐small‐cell lung cancer after curative resection with or without induction/adjuvant chemotherapy. Interact Cardiovasc Thorac Surg. 2013;16:166–72. 23143203 10.1093/icvts/ivs450 PMC3548532

[3] Shimada Y , Saji H , Yoshida K , Kakihana M , Honda H , Nomura M , et al. Prognostic factors and the significance of treatment after recurrence in completely resected stage I non‐small cell lung cancer. Chest. 2013;143:1626–34. 23348916 10.1378/chest.12-1717

[4] Takenaka T , Takenoyama M , Yamaguchi M , Toyozawa R , Inamasu E , Kojo M , et al. Impact of the epidermal growth factor receptor mutation status on the post‐recurrence survival of patients with surgically resected non‐small‐cell lung cancer. Eur J Cardiothorac Surg. 2015;47:550–5. 24894095 10.1093/ejcts/ezu227

[5] Sonoda D , Matsuura Y , Kondo Y , Ichinose J , Nakao M , Ninomiya H , et al. Characteristics of surgically resected non‐small cell lung cancer patients with post‐recurrence cure. Thorac Cancer. 2020;11:3280–8. 32961037 10.1111/1759-7714.13669 PMC7605994

[6] Kenmotsu H , Yamamoto N , Yamanaka T , Yoshiya K , Takahashi T , Ueno T , et al. Randomized phase III study of pemetrexed plus cisplatin versus vinorelbine plus cisplatin for completely resected stage II to IIIA nonsquamous non‐small‐cell lung cancer. J Clin Oncol. 2020;38:2187–96. 32407216 10.1200/JCO.19.02674

[7] Xu ST , Xi JJ , Zhong WZ , Mao WM , Wu L , Shen Y , et al. The unique spatial‐temporal treatment failure patterns of adjuvant gefitinib therapy: a post hoc analysis of the ADJUVANT trial (CTONG 1104). J Thorac Oncol. 2019;14:503–12. 30521970 10.1016/j.jtho.2018.11.020

[8] Wu YL , Tsuboi M , He J , John T , Grohe C , Majem M , et al. Osimertinib in resected *EGFR*‐mutated non‐small‐cell lung cancer. N Engl J Med. 2020;383:1711–23. 32955177 10.1056/NEJMoa2027071

[9] NCCN . “Guidelines for Non‐Small Cell Lung Cancer.”. https://www.nccn.org/guidelines/guidelines‐detail?category=1&id=1450 (Accessed 4 Jun 2021).

[10] Hishida T , Yoshida J , Aokage K , Nagai K , Tsuboi M . Postoperative oligo‐recurrence of non‐small‐cell lung cancer: clinical features and survival. Eur J Cardiothorac Surg. 2016;49:847–53. 26201958 10.1093/ejcts/ezv249

[11] Yano T , Okamoto T , Haro A , Fukuyama S , Yoshida T , Kohno M , et al. Local treatment of oligometastatic recurrence in patients with resected non‐small cell lung cancer. Lung Cancer. 2013;82:431–5. 24113550 10.1016/j.lungcan.2013.08.006

[12] Matsuguma H , Nakahara R , Wakamatsu I , Kishikawa T , Sugiyama T , Nakamura Y , et al. Definitive local therapy for oligo‐recurrence in patients with completely resected non‐small cell lung cancer. Am J Clin Oncol. 2020;43:210–7. 31850917 10.1097/COC.0000000000000656

[13] Niibe Y , Nishimura T , Inoue T , Karasawa K , Shioyama Y , Jingu K , et al. Oligo‐recurrence predicts favorable prognosis of brain‐only oligometastases in patients with non‐small cell lung cancer treated with stereotactic radiosurgery or stereotactic radiotherapy: a multi‐institutional study of 61 subjects. BMC Cancer. 2016;19(16):659. 10.1186/s12885-016-2680-8 PMC4992231 27542716

[14] Seol KH , Lee JE , Cho JY , Lee DH , Seok Y , Kang MK . Salvage radiotherapy for regional lymph node oligo‐recurrence after radical surgery of non‐small cell lung cancer. Thorac Cancer. 2017;8:620–9. 28906073 10.1111/1759-7714.12497 PMC5668518

[15] Han SJ , Cho S , Yum S , Kim K , Jheon S . Surgical treatment of pulmonary oligorecurrence after curative resection for non‐small‐cell lung cancer. Interact Cardiovasc Thorac Surg. 2020;30:18–23. 31539023 10.1093/icvts/ivz221

[16] Aoki S , Yamashita H , Takahashi W , Nawa K , Ota T , Imae T , et al. Salvage stereotactic body radiotherapy for post‐operative oligo‐recurrence of non‐small cell lung cancer: a single‐institution analysis of 59 patients. Oncol Lett. 2020;19:2695–704. 32218820 10.3892/ol.2020.11407 PMC7068670

[17] Sharma SV , Bell DW , Settleman J , Haber DA . Epidermal growth factor receptor mutations in lung cancer. Nat Rev Cancer. 2007;7:169–81. 17318210 10.1038/nrc2088

[18] Sholl LM , Aisner DL , Varella‐Garcia M , Berry LD , Dias‐Santagata D , Wistuba II , et al. Multi‐institutional oncogenic driver mutation analysis in lung adenocarcinoma: the lung cancer mutation consortium experience. J Thorac Oncol. 2015;10:768–77. 25738220 10.1097/JTO.0000000000000516 PMC4410843

[19] Tan AC , Tan DSW . Targeted therapies for lung cancer patients with oncogenic driver molecular alterations. J Clin Oncol. 2022;40:611–25. 34985916 10.1200/JCO.21.01626

[20] World Health Organization . Histological typing of lung and pleural Tumors. 3rd ed. Geneva: Springer‐Verlag; 1999.

[21] Travis WD , Brambilla E , Nicholson AG , Yatabe Y , Austin JHM , Beasley MB , et al. The 2015 World Health Organization classification of lung tumors: impact of genetic, clinical and radiologic advances since the 2004 classification. J Thorac Oncol. 2015;10:1243–60. 26291008 10.1097/JTO.0000000000000630

[22] Martini N , Melamed MR . Multiple primary lung cancers. J Thorac Cardiovasc Surg. 1975;70:606–12. 170482

[23] Ramalingam SS , Vansteenkiste J , Planchard D , Cho BC , Gray JE , Ohe Y , et al. Overall survival with osimertinib in untreated, *EGFR*‐mutated advanced NSCLC. N Engl J Med. 2020;382:41–50. 31751012 10.1056/NEJMoa1913662

[24] Takenaka T , Takenoyama M , Toyozawa R , Inamasu E , Yoshida T , Toyokawa G , et al. Concurrent chemoradiotherapy for patients with postoperative recurrence of surgically resected non‐small‐cell lung cancer. Clin Lung Cancer. 2015;16:51–6. 25038000 10.1016/j.cllc.2014.06.001

