# Supplementary material for: Is radical local therapy effective in postoperative recurrent EGFR ‐mutated non‐small cell lung cancer?
Source: Thorac Cancer. 2023 May 4;14(18):1660–7. doi: 10.1111/1759-7714.14911 (PMC10290912; doi:10.1111/1759-7714.14911)
Supplement: Supplementary file 1 — Figure S1. Consort diagram of the study. [file TCA-14-1660-s001.pdf]

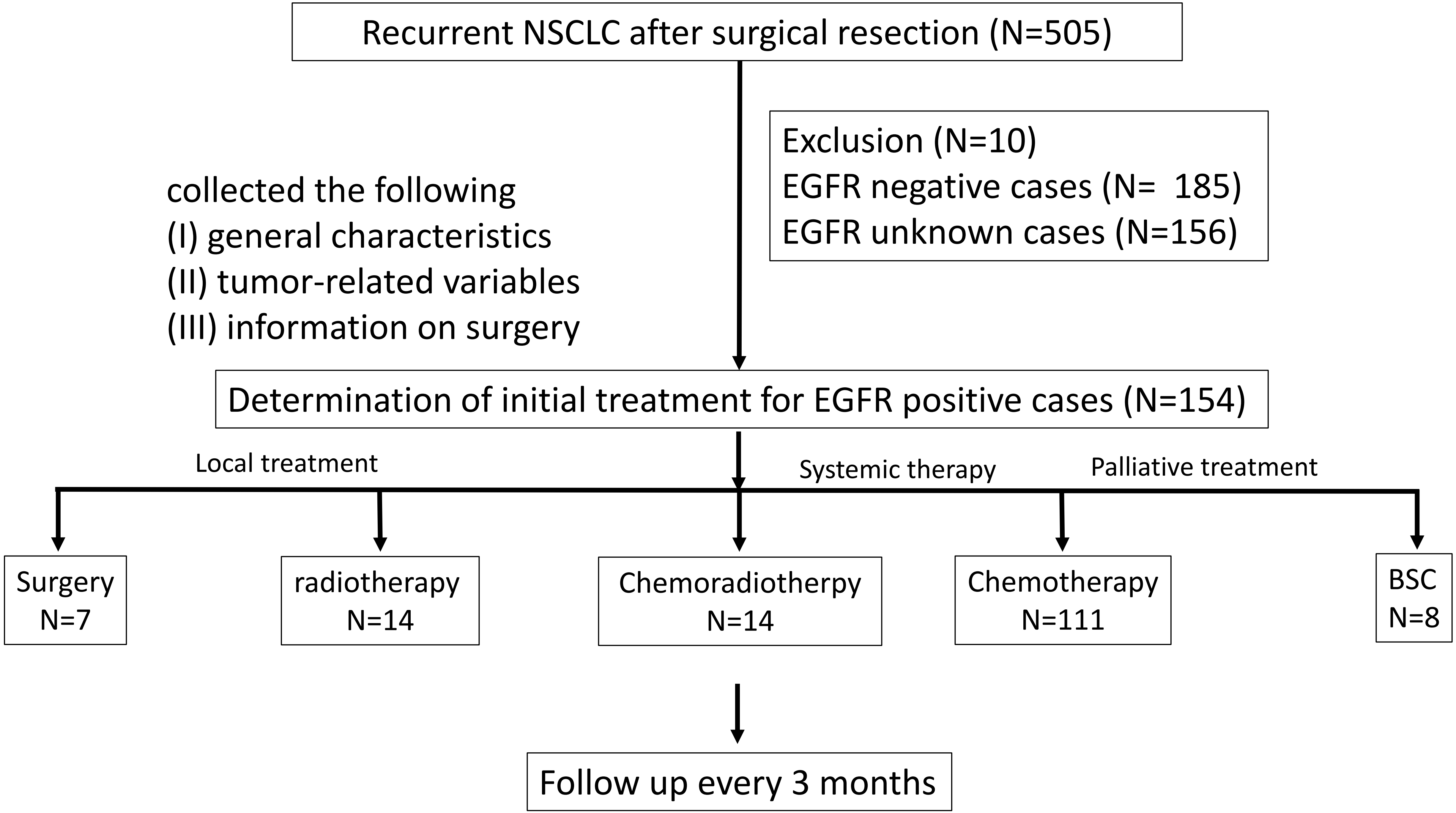
Recurrent NSCLC after surgical resection (N=505)
Exclusion (N=10)
EGFR negative cases (N= 185)
EGFR unknown cases (N=156)
collected the following
(I) general characteristics
(II) tumor-related variables
(III) information on surgery
Determination of initial treatment for EGFR positive cases (N=154)
Local treatment
Systemic therapy
Palliative treatment
Surgery N=7
radiotherapy N=14
Chemoradiotherpy N=14
Chemotherapy N=111
BSC N=8
Follow up every 3 months
